# Vasorelaxin: A Novel Arterial Smooth Muscle-Relaxing Eicosapeptide from the Skin Secretion of the Chinese Piebald Odorous Frog (*Odorrana schmackeri*)

**DOI:** 10.1371/journal.pone.0055739

**Published:** 2013-02-06

**Authors:** Yuxin Wu, Lei Wang, Chen Lin, Yan Lin, Mei Zhou, Liang Chen, Brian Connolly, Yingqi Zhang, Tianbao Chen, Chris Shaw

**Affiliations:** 1 Natural Drug Discovery Group, School of Pharmacy, Queen’s University, Belfast, Northern Ireland, United Kingdom; 2 Tangshan Gongren Hospital, No.27, Wenhua Road, Tangshan City, Hebei Province, China; University of Giessen Lung Center, Germany

## Abstract

The defensive skin secretions of amphibians are a rich resource for the discovery of novel, bioactive peptides. Here we report the identification of a novel vascular smooth muscle-relaxing peptide, named vasorelaxin, from the skin secretion of the Chinese piebald odorous frog, *Odorrana schmackeri*. Vasorelaxin consists of 20 amino acid residues, SRVVKCSGFRPGSPDSREFC, with a disulfide-bridge between Cys-6 and Cys-20. The structure of its biosynthetic precursor was deduced from cloned skin cDNA and consists of 67 amino acid residues encoding a single copy of vasorelaxin (vasorelaxin, accession number: HE860494). Synthetic vasorelaxin caused a profound relaxation of rat arterial smooth muscle with an EC_50_ of 6.76 nM.

## Introduction

The failure of the chemical approach to expand and replenish the drug pipelines of the pharmaceutical industry has led to a renaissance in natural drug discovery, especially in so-called “biologics” [1], [2]. Venoms and defensive secretions represent libraries of “smart” molecules that have been sorted and perfected by eons of natural selection in the biosphere and, as a consequence of this, are generally precision-targeted and highly-potent [3]–[6]. While research on reptile venoms has produced natural peptide leads fundamental to the development of angiotensin-converting enzyme (ACE)-inhibitors for the treatment of hypertension [7] and more recently, exenatide, for the treatment of type-2 diabetes [8], the focus on the peptidomes of amphibian skin defensive secretions is of more recent occurrence. However, focused studies on the bioactive components of these secretions have shown unequivocally, that they are unique sources of a plethora of potent biologically-active molecules that act on an array of endogenous mammalian/human molecular targets, many of which are disease-associated [3]–[8]. These amphibian skin-derived molecules are thought to represent the fundamental components of an intrinsic survival mechanism that has obviously served amphibians well for many millions of years. While the antimicrobial peptides, which constitute an innate immune defense system, defend the amphibian against microbial invasion, the pharmacologically-active peptides defend the amphibian against predators by interfering with their endogenous sensory or signalling systems [3], [9], [10]. Families of structurally-related peptides which perform the latter function include the bradykinins, tachykinins, ceruleins and opioids [3], [9], [10]. However, with increasingly focused efforts directed towards amphibian skin peptidome characterization, prototypes of novel families have been recently-discovered that include kassorins [11], limnonectins, [12], sauvatides [13] and kasstasin [14]. The latter peptide is a vasoconstrictor peptide as determined by its high potency in contracting rat tail artery smooth muscle.

Here, we report the isolation, structural, and preliminary pharmacological characterization of a prototype of a novel class of potent vasodilatory peptide by use of the same bioassay of skin secretion fractions from the Chinese piebald odorous frog, *Odorrana schmackeri.* This eicosapeptide (20 amino acid residues) was named vasorelaxin in accordance with its biological activity.

## Materials and Methods

### Specimen Biodata and Secretion Harvesting

*Odorrana schmackeri* (n = 3, 5–7 cm snout-to-vent length, sex undetermined) were captured during expeditions to the Wuyi Mountain river of Fujian Province in the People’s Republic of China. Permission to sample frogs was obtained from collaborators in the Institute of Biotechnology of Fuzhou University. Sampling of skin secretion was performed by MZ under UK Animal (Scientific Procedures) Act 1986, project licence PPL 2694, issued by the Department of Health, Social Services and Public Safety, Northern Ireland. Procedures had been vetted by the IACUC of Queen's University Belfast, and approved.

All frogs were adults and secretion harvesting was performed in the field after which frogs were released. Skin secretion was obtained from the dorsal skin using gentle transdermal electrical stimulation as previously described [15]. The stimulated secretions were washed from the skin using deionized water and divided into either 0.2% v/v aqueous trifluoroacetic acid for subsequent peptide characterization, or into cell lysis/mRNA stabilization buffer (Dynal) for subsequent cDNA library construction.

### Reverse-phase HPLC Fractionation

The acidified skin secretion washings were clarified of microparticulates by centrifugation. The clear supernatant was then subjected to reverse-phase HPLC fractionation using a Cecil Adept Binary HPLC system fitted with a Jupiter semi-preparative C-5 column (30×1 cm). This was eluted with a linear gradient formed from 0.05/99.5 (v/v) TFA/water to 0.05/19.95/80.0 (v/v/v) TFA/water/acetonitrile in 240 min at a flow rate of 1 ml/min. Fractions (1 ml) were collected at minute intervals and the effluent absorbance was continuously monitored at λ214 nm. Samples (100 µl) were removed from each fraction in triplicate, lyophilized and stored at –20°C prior to arterial smooth muscle pharmacological analysis.

### Bioactivity Screening Using Rat Tail Artery Smooth Muscle

Adult male Wistar rats, weighing 200–250 g, were euthanized by animal unit staff using asphyxiation with CO_2_ followed by cervical dislocation. Tissues were dissected from cadavers by MZ under UK Animal (Scientific Procedures) Act 1986, personal licence PIL 1309, issued by the Department of Health, Social Services and Public Safety, Northern Ireland. Procedures had been vetted by the IACUC of Queen's University Belfast.

The animals were laid on their dorsal surfaces, following which the tail skin was removed. The tail artery vascular bed was identified and moistened with Krebs’ solution (118 mM NaCl, 4.7 mM KCl, 25 mM NaHCO_3_, 1.15 mM NaH_2_PO_4_, 2.5 mM CaCl_2_, 1.1 mM MgCl_2_ and 5.6 mM glucose) that had been equilibrated with 95% O_2_/5%CO_2_. The membrane and the connective tissue beneath the main artery were carefully removed. The proximal region of the tail artery was excised and immediately placed in ice-cold Kreb’s solution. Two millimetre-wide rings of artery were cut and mounted on a transducer prior to placing in 2 ml organ baths containing Kreb’s solution flowing through at 2 ml/min and maintained at 37°C with constant bubbling of 95% O_2_,/5%CO_2_. Muscle rings were pre-contracted with 10^−5^ M phenylephrine and equilibrated for 1 h before experimental procedures were initiated. Samples of sequential reverse phase HPLC fractions were reconstituted in the same volume of Kreb’s solution before screening for smooth muscle activity.

### Structural Analyses and Chemical Synthesis

The reverse phase HPLC fraction that possessed the highest arterial smooth muscle relaxing activity as determined by bioassay, was initially analyzed using matrix-assisted, laser desorption, ionization, time-of-flight mass spectrometry (MALDI-TOF MS) on a linear time-of-flight Voyager DE mass spectrometer (Perseptive Biosystems, MA, USA) in positive detection mode using α-cyano-4-hydroxycinnamic acid as the matrix. Internal mass calibration of the instrument with known standards established the accuracy of mass determination as ±0.1%. After determination of sample purity and molecular mass, the peptide was subjected to MS/MS fragmentation and *de novo* sequence analysis using an LCQ-Fleet™ electrospray ion-trap mass spectrometer.

Once the unequivocal primary structure of the vasorelaxing peptide had been established, it was chemically-synthesized using solid-phase Fmoc chemistry by means of a PS3 automated solid-phase peptide synthesizer (Protein Technologies, Inc., AZ, USA). Following cleavage from the resin and deprotection, the synthetic peptide was analyzed by both reverse phase HPLC and MALDI-TOF mass spectrometry to establish degree of purity and authenticity of structure. For repeat pharmacological experiments, the purified synthetic peptide was standardized by means of acid hydrolysis of a known gravimetric quantity, followed by amino acid analysis using an Applied Biosystems PTH-amino acid analyzer.

### Molecular Cloning of cDNA Encoding the Biosynthetic Precursor of the Novel Vasorelaxant Peptide from the Skin Secretion-derived cDNA Library

Polyadenylated mRNA was isolated from the stabilization buffer using magnetic oligo-dT beads as described by the manufacturer (Dynal Biotech, UK) and reverse-transcribed. The cDNA was subjected to 3′ and 5′ - RACE procedures to obtain full-length prepro-peptide nucleic acid sequence data using a SMART-RACE kit (Clontech UK) essentially as described by the manufacturer. Briefly, the 3′-RACE reactions employed an NUP primer (supplied with the kit) and a degenerate sense primer (S; 5′- GTIGTIAARTGYWSIGGITT -3′) that was complementary to the internal amino acid sequence, -VVKCSGF -, of the vasorelaxing peptide. PCR products were gel-purified and cloned using a pGEM-T vector system and sequenced. According to the 3′-RACE product sequence, a specific antisense primer was designed to a conserved site within the 3′-non-translated region. The 5′-RACE reaction was performed by using this antisense primer, designated AS (5′-CCACATCAGATGATTTCCAATCAAGAT-3′), in conjunction with the NUP RACE primer. Generated PCR products were gel-purified, cloned, and sequenced as described previously [11]–[14].

### Pharmacological Assay of the Synthetic Novel Peptide Using the Rat Tail Artery Smooth Muscle Preparation

Solutions of synthetic novel peptide and bradykinin, ranging in concentration from 10^−11^ to 10^−5^ M, were prepared in Kreb’s buffer and were used to construct separate dose–response curves. These were added to the arterial smooth muscle preparations in increasing concentrations with 5 min washes and 5 min equilibration periods between each dose. Each peptide concentration was applied to a minimum of five different preparations. Changes in tension of the artery muscle rings were recorded and amplified through force transducers connected to a PowerLab System (AD Instruments Pty Ltd.). Data were analyzed to obtain the mean and standard error of responses by Student’s *t* test and dose–response curves were constructed using a best-fit algorithm through the data analysis package provided. Responses were plotted as negative changes in grams of tension against final molar concentrations of peptides present in the organ baths.

### Preliminary Studies on the Mechanisms of Action of the Novel Vasorelaxing Peptide

In order to determine if the action of the novel vasorelaxing peptide was due to inherent cytotoxicity, two different experiments were performed. In the first series of experiments, cultured human microvessel endothelial cells (HMECs) were incubated for 24 h with concentrations of synthetic novel peptide ranging from 10^−11^ M to 10^−5^ M. Cell proliferation/viability was measured using the MTT assay which has been described in detail previously [16]. In a second series of experiments, a single maximal arterial smooth muscle relaxing dose (10^−7^ M) of novel peptide was added to the arterial smooth muscle preparation and the response recorded. Following washout and a re-equilibration period of 10 min, this dose was repeated 4 times in sequence with washouts in between.

Although the method of preparation of the arterial smooth muscle rings essentially denudes the endothelial lining, as evidenced by their responsiveness to phenylephrine [17], the possible contribution of endothelial-derived NO to the observed smooth muscle relaxation, was examined. Arterial smooth muscle preparations were pre-treated for 20 min with the eNOS inhibitor, *N* ^5^-(1-iminoethyl)-L-ornithine dihydrochloride (L-NIO, Tocris Biosciences), at 500 nM, prior to construction of a dose-response curve with the novel peptide from 10^−11^ M to 10^−5^ M.

In order to determine if the vasorelaxant action of the novel peptide on arterial smooth muscle involved interaction with bradykinin B2 receptors, dose-response curves of the novel peptide at concentrations between 10^−11^ M and 10^−5^ M, were constructed in the presence and absence of the specific bradykinin B2 receptor antagonist, HOE-140 (Sigma Aldrich), at a single concentration of 3×10^−7^ M.

Data from each of these experiments were analyzed by 2-way ANOVA.

## Results

### Identification and Structural Characterization of Vasorelaxin

**Figure 1:**
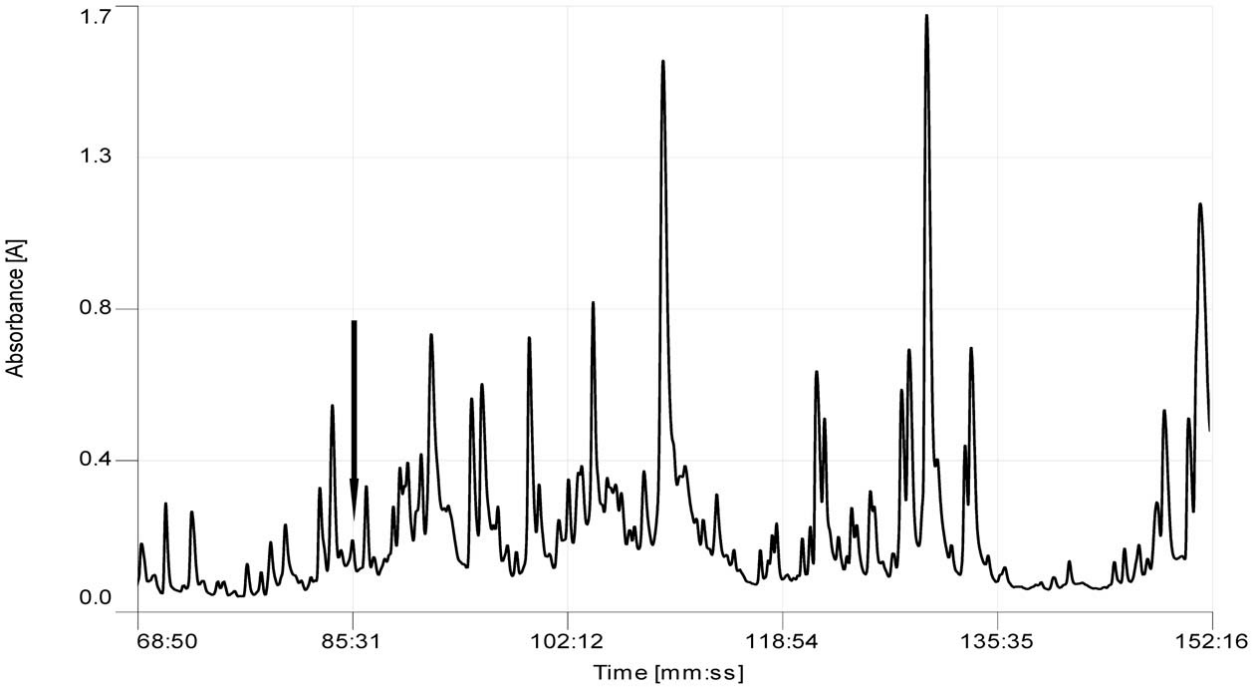
Region of reverse phase HPLC chromatogram of *Odorrana schmackeri* skin secretion. Elution position/retention time of the vasorelaxant peptide (vasorelaxin) is indicated (arrow).

**Figure 2:**
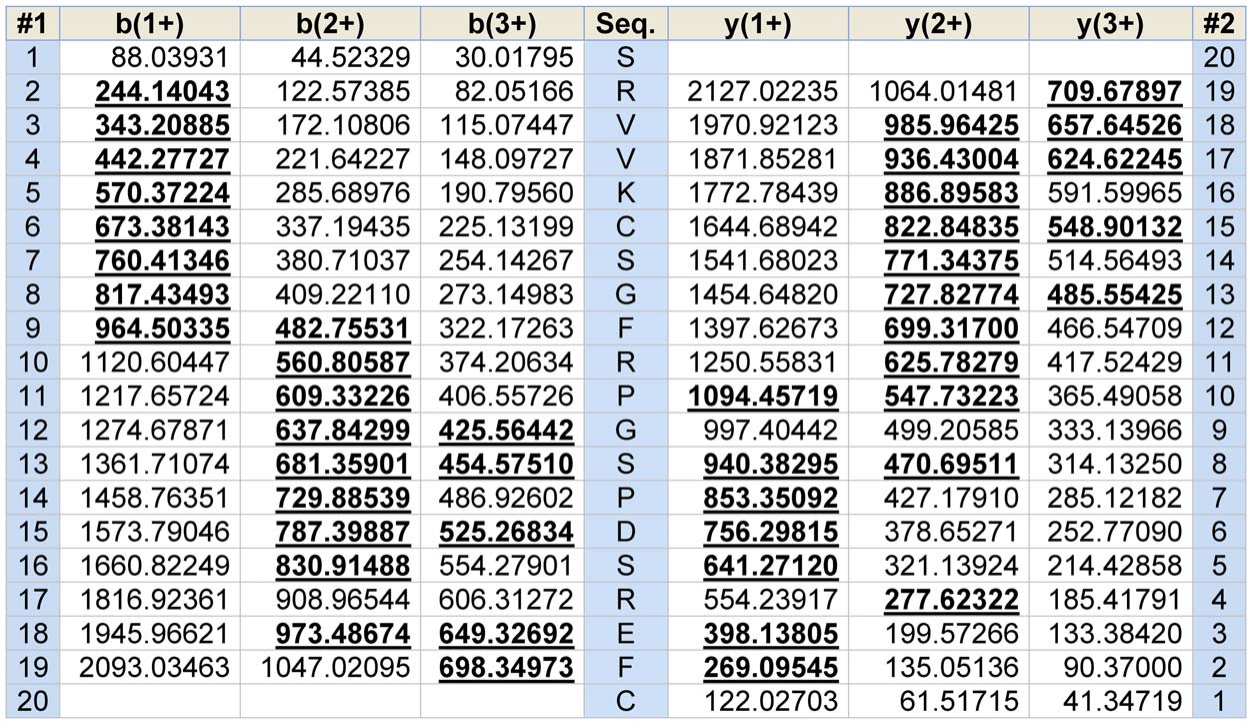
MS/MS fragmentation of vasorelaxin. Theoretical (normal typeface) and observed (bold typeface) *b*-ions and *y*-ions are shown.

Following screening of reverse phase HPLC fractions of *Odorrana schmackeri* skin secretion for vasoactives, using the rat tail arterial smooth muscle preparation, a major relaxant activity was found in fraction #85, coincident with a small but discrete absorbance peak as shown in Figure 1. MALDI-TOF MS analysis of this fraction showed that it contained a single major peptide with an m/z of 2213.75 (M+H)^+^. MS/MS fragmentation analysis of this peptide using an LCQ Fleet™ electrospray ion-trap mass spectrometer, established the primary structure of this peptide through use of *de novo* sequencing software as illustrated in Figure 2. Interrogation of contemporary protein/peptide databases indicated no structural similarity with any known vasoactive peptide or protein. By nature of its novel structure and potent vasorelaxing activity, this peptide was named, vasorelaxin. Vasorelaxin thus represents the prototype of a novel class of vasoactive peptide from amphibian skin.

### Molecular Cloning of Vasorelaxin Biosynthetic Precursor-encoding cDNA

**Figure 3:**
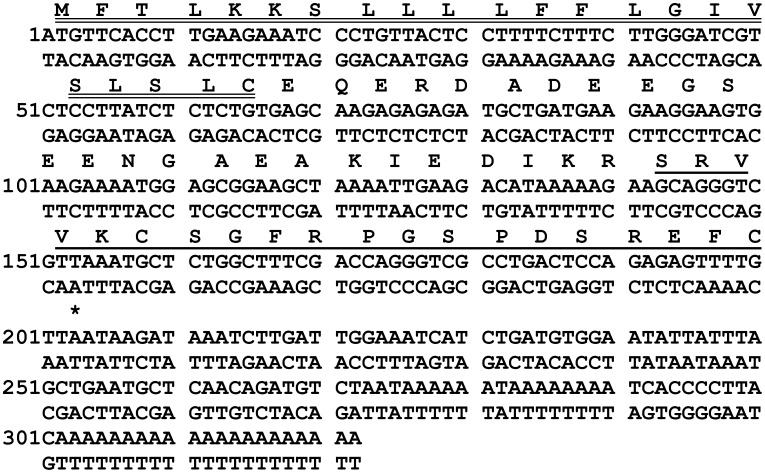
Nucleotide and translated open-reading frame amino acid sequence of the cloned cDNA encoding the biosynthetic precursor of vasorelaxin. The putative signal peptide is double-underlined, the mature vasorelaxin peptide is single-underlined and the stop codon is indicated with an asterisk.

A cDNA, whose open-reading frame encoded the biosynthetic precursor of vasorelaxin, was successfully and repeatedly (>25×) cloned from the skin secretion-derived cDNA library, using the RACE-PCR protocol described. The open-reading frame of the cDNA consisted of 67 amino acid residues, beginning with a putative hydrophobic signal peptide of 22 residues, followed by an acidic residue-rich intervening peptide of 23 residues, a typical dibasic residue propeptide convertase processing site (-Lys-Arg-, -K-R-) and terminating in a single domain encoding the mature vasorelaxin peptide (Figure 3). An NCBI-BLAST search revealed no structural similarity to any vasoactive peptide or protein in the database (vasorelaxin, accession number: HE860494). However, vasorelaxin displayed some sequence similarity with an antimicrobial peptide, lividin-9 (accession number ACA81701, unpublished), but was found to be devoid of such activity against model Gram-positive (*Staphylococcus aureus –* NCTC.10788) and Gram-negative (*Escherichia coli –* NCTC.10418) bacteria and the yeast (*Candida albicans –* NCTC.1467), at concentrations up to and exceeding 500 µM (data not shown).

### Rat Tail Artery Smooth Muscle Pharmacology

**Figure 4:**
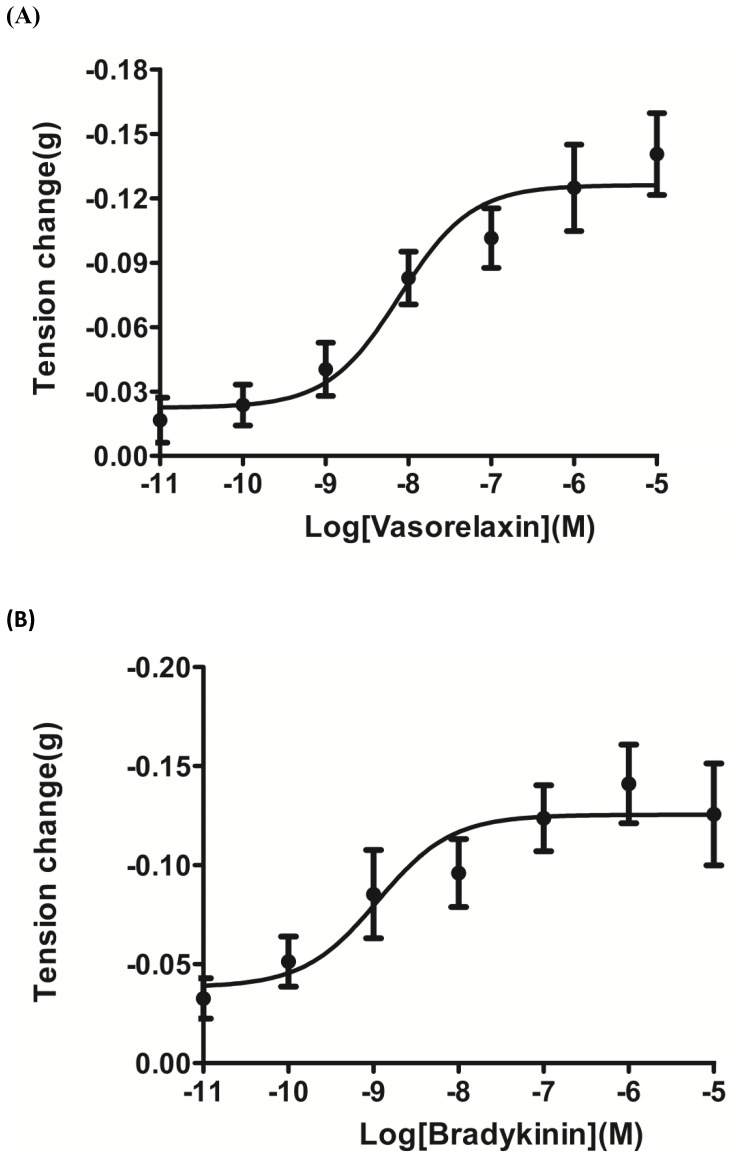
Comparative pharmacology of synthetic vasorelaxin and bradykinin. Relaxation dose-response curves of (**A**) synthetic vasorelaxin and (**B**) synthetic bradykinin obtained using rat tail artery smooth muscle preparations. Each data-point represents the mean and standard error of responses from five different preparations in each case.

Vasorelaxin was successfully synthesized by solid-phase Fmoc methodology and purified using semi-preparative LC/MS to >95% purity. The peptide induced a dose-dependent relaxation of rat arterial smooth muscle (Figure 4A). Bradykinin, known to be an endogenous relaxing factor for rat tail artery smooth muscle, was included in a series of parallel experiments for the purposes of comparison (Figure 4B). Vasorelaxin (EC_50_ = 6.76 nM) was found to have a similar potency to bradykinin in the low nanomolar range (EC_50_ = 1.12 nM).

### Preliminary Studies on the Mechanisms of Action of Vasorelaxin

**Figure 5:**
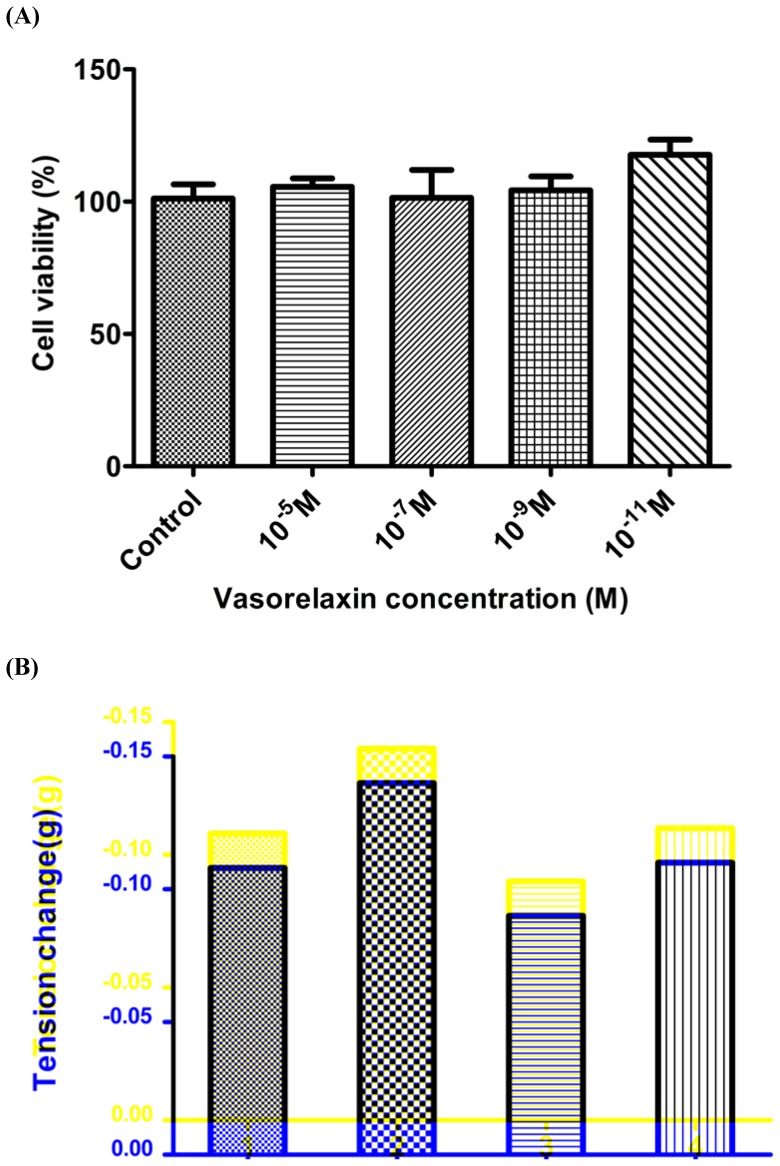
Assessment of putative cytotoxicity of vasolrelaxin. (**A**) The effect of synthetic vasorelaxin (10^−5^ M to 10^−11^ M) on the growth of human microvessel endothelial cells (HMECs) as assessed by the MTT assay, following a 24 h incubation at 37°C. Each column represents the mean±SEM of 8 replicates. There were no significant effects observed in growth compared to controls (10^−5^ M – p = 0.588, 10^−7^ M, p = 0.977, 10^−9^ M – p = 0.719, 10^−11^ M – p = 0.071; One-way ANOVA). (**B**) The effect of 4 sequential additions of a single dose (10^−7^ M) of vasorelaxin indicating continued responsiveness of the preparation.

Vasorelaxin was incubated with human microvessel endothelial cells (HMECs) in a range of concentrations for 24 h and the growth/viability of cells was assessed using the MTT assay. The results (Figure 5A) indicated that vasorelaxin lacked cytotoxic effects in this assay over the concentration range tested even when present at significantly higher molar excess (10^−5^ M) than effective arterial smooth muscle relaxant concentrations. In addition, sequential addition of the same doses of vasorelaxin to a single arterial smooth muscle preparation did not significantly alter its responsiveness indicative of a specific rather than cytotoxic mode of action (Figure 5B).

**Figure 6:**
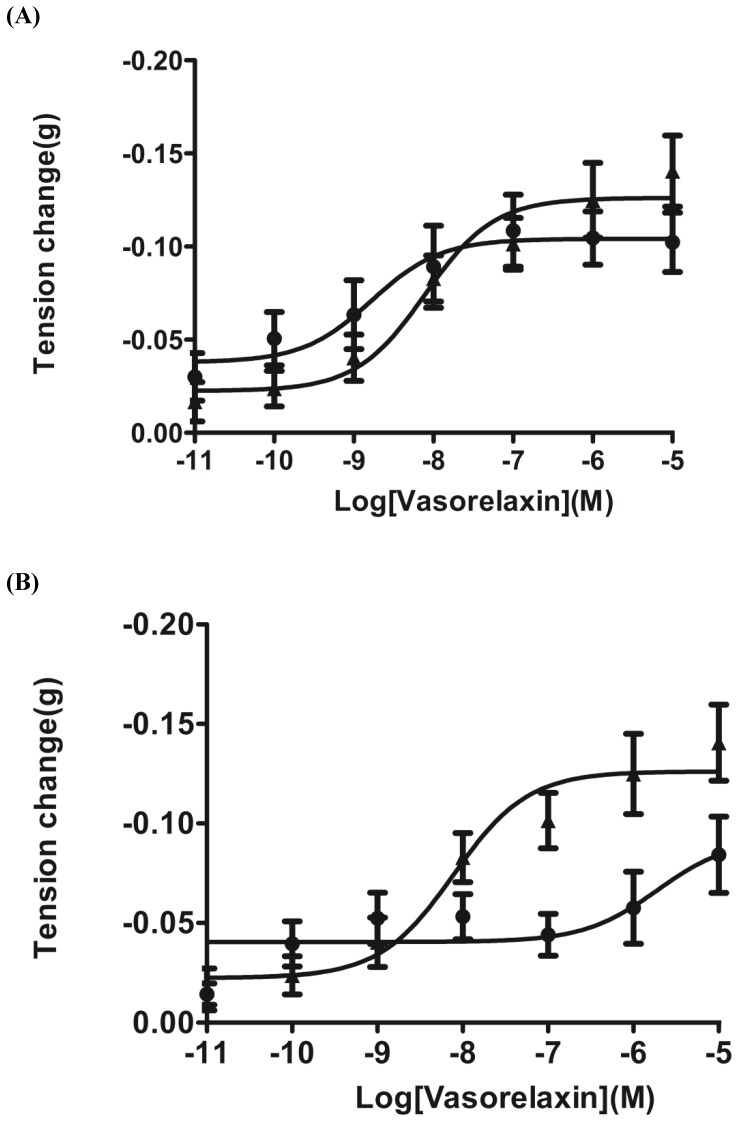
Assessment of the effects of eNOS and bradykinin B2 receptor inhibition on the smooth muscle effects of vasorelaxin. (**A**) Dose-response curves of vasorelaxin on rat tail artery smooth muscle preparations in the absence (▴) or presence (▪) of the eNOS inhibitor, L-NIO. Data points represent the mean ±SEM of 6 replicates. No significant difference (P value 0.7609, 2-way ANOVA) was found for the effect of this inhibitor on the activity of vasorelaxin. (B) Dose-response curves of vasorelaxin on rat tail artery smooth muscle preparations in the absence (▴) or presence (▪) of the bradykinin B2 receptor antagonist, HOE-140. Data points represent the mean ±SEM of 6 replicates. A significant difference (P value 0.0012, 2-way ANOVA) was found in the responsiveness of preparations to vasorelaxin.

Pre-treatment of preparations with the eNOS inhibitor, L-NIO, prior to construction of a vasorelaxin dose-response curve, had no significant effects on tissue responsiveness (P value 0.7609, 2-way ANOVA) (Figure 6A), indicating that NO was unlikely to be involved in induction of the vasorelaxant effects.

In contrast, pre-treatment of preparations with the specific bradykinin B2 receptor antagonist, HOE-140, prior to construction of a vasorelaxin dose-response curve, had a significant inhibition effect (P value 0.0012, 2-way ANOVA) on the vasorelaxation induced by vasorelaxin between the concentrations of 10^−8^ and 10^−5^ M (Figure 6B).

## Discussion

Amphibian skin secretions continue to be validated as unique sources of novel biologically-active peptides [3], [9], [10]. The defensive peptides of such secretions fall into three broad categories based upon their originally-identified biological effects and these are antimicrobials [18], protease inhibitors [19]–[21] and those with profound pharmacological effects on endogenous mammalian physiological systems [22]–[25]. The latter group are the most structurally-diverse, often share identical bioactive core sequences with endogenous mammalian peptides and act through the same G protein-coupled receptors (GPCRs) [22]–[25]. Some, such as bradykinin and thyrotropin-releasing hormone (TRH), are identical in primary structure to respective mammalian counterparts, but occur in concentrations that are vastly in excess of those found within mammalian tissues [24], [25]. The pharmacologically-active peptides of amphibian skin can generally be regarded as site-substituted analogs of endogenous vertebrate regulatory peptides and thus can be classified into known families of such, examples being the bradykinins, tachykinins and bombesins [22]–[24]. However, with an increasing focus on the study of amphibian skin secretion peptidomes and the application of modern analytical technologies, there are ever-growing numbers of unique peptides that are being reported from this source. Here, we describe one such peptide, named vasorelaxin, isolated from skin secretion of the Chinese piebald odorous frog, *Odorrana schmackeri*, by nature of its relaxation effect on rat tail artery smooth muscle. A synthetic replicate of vasorelaxin was used to determine the potency of the dilatory effect on this preparation and it was found to be of similar potency to bradykinin. Since bradykinin mediates this effect through interaction with specific cognate receptors, it is not unlikely that the similar potency of vasorelaxin, in this respect, would suggest that it does the same. Vasorelaxin exhibits little similarity, in terms of primary structure, with any peptide known to possess this property or indeed, with any peptide known to act, as does bradykinin, with cognate GPCRs. Although it would be tempting to suggest that vasorelaxin is a cognate ligand for an orphan GPCR, it may well be that lack of primary structural similarity hides the possibility that it is a tertiary structural mimic of a known ligand for a known receptor. However, the low nanomolar potency of this peptide would tend to rule out a non-specific effect as this is well within the range of interaction/activation of known peptide ligand/GPCR systems within biological preparations [22]–[25]. For these reasons, we tested the effect of a well-established bradykinin B2 receptor antagonist, HOE-140, on the arterial smooth muscle relaxing ability of vasorelaxin and were surprised to observe a significant reduction. Although these data might imply that vasorelaxin acts through the bradykinin B2 receptor, further experiments will be necessary to further address this aspect of the mechanism of action more fully. It could be for instance, that HOE-140 can antagonize more GPCRs than the bradykinin B2 receptor in a manner similar to that of the NK1 tachykinin receptor substance P antagonist, [D-Arg^1^, D-Phe^5^, D-Trp^7,9^, Leu^11^]-substance P, that also acts as a potent antagonist of bombesin at GRP receptors [26]. Many vasodilators act via a common final pathway that involves stimulation of endothelium nitric oxide synthase (eNOS) with subsequent release of NO – the final effector molecule that causes smooth muscle relaxation [27]. Pre-treatment of arterial smooth muscle preparations with a specific eNOS inhibitor, failed to cause significant effects on the dose-responsive relaxation of vasorelaxin indicating that this effect is unlikely to involve the action of NO. These data would suggest that vasorelaxin has direct effects on arterial smooth muscle cells and is non-toxic to such thus paving the way for more extensive systematic pharmacological characterization of its mode of action.

Amphibian skin thus continues to provide unique peptide structures with discrete biological potencies that rival or exceed those of endogenous regulatory peptides. Some of these molecules may constitute leads in the design of novel drugs for a plethora of therapeutic end-points.

## References

[1] Gale EA (2001) Lessons from the glitazones: a story of drug development. Lancet 357: 1870–1875. 11410214 10.1016/S0140-6736(00)04960-6

[2] Paterson I, Anderson EA (2005) Chemistry. The renaissance of natural products as drug candidates. Science 310: 451–453. 16239465 10.1126/science.1116364

[3] Lazarus LH, Attila M (1993) The toad, ugly and venomous, wears yet a precious jewel in his skin. Prog Neurobiol 41: 473–507. 8210414 10.1016/0301-0082(93)90027-p

[4] Raufman JP (1996) Bioactive peptides from lizard venoms. Regul Pept 61: 1–18. 8701022 10.1016/0167-0115(96)00135-8

[5] Pal SK, Gomes A, Dasgupta SC, Gomes A (2002) Snake venom as therapeutic agents: from toxin to drug development. Indian J Exp Biol 40: 1353–1358. 12974396

[6] Srinivasan KN, Gopalakrishnakone P, Tan PT, Chew KC, Cheng B, et al (2002) SCORPION, a molecular database of scorpion toxins. Toxicon 40: 23–31. 11602275 10.1016/s0041-0101(01)00182-9

[7] Patlak M (2004) From viper's venom to drug design: treating hypertension. FASEB J 18: 421. 15003987 10.1096/fj.03-1398bkt

[8] Davidson MB, Bate, Kirkpatrick P (2005) Exenatide. Nat Rev Drug Discov 4: 713–714. 16178120 10.1038/nrd1828

[9] Erspamer V (1994) Bioactive secretions of the integument. In: Heatwole H, Barthalmus GT, editors, Amphibian biology, The integument, vol. 1. Beatty and Sons, Chipping Norton, Surrey. 179–350.

[10] Bevins CL, Zasloff M (1990) Peptides from frog skin. Ann Rev Biochem 59: 395–414. 2197979 10.1146/annurev.bi.59.070190.002143

[11] Chen H, Wang L, Zeller M, Hornshaw M, Wu Y, et al (2011) Kassorins: novel innate immune system peptides from skin secretions of the African hyperoliid frogs, *Kassina maculata* and *Kassina senegalensis* . Mol Immunol 48: 442–451. 21040978 10.1016/j.molimm.2010.09.018

[12] Wu Y, Wang L, Zhou M, Ma C, Chen X, et al (2011) Limnonectins: a new class of antimicrobial peptides from the skin secretion of the Fujian large-headed frog (*Limnonectes fujianensis*). Biochimie 93: 981–987. 21396976 10.1016/j.biochi.2011.03.003

[13] Wang L, Zhou M, Zhou Z, Chen T, Walker B, et al (2009) Sauvatide–a novel amidated myotropic decapeptide from the skin secretion of the waxy monkey frog, *Phyllomedusa sauvagei*. Biochem. Biophys Res Commun 383: 240–244. 10.1016/j.bbrc.2009.04.003 19358833

[14] Li X, Feng W, Zhou M, Ma C, Chen T, et al (2011) Kasstasin: A novel potent vasoconstrictor peptide from the skin secretion of the African red-legged running frog, *Kassina maculata* . Biochimie 93: 1537–1542. 21624426 10.1016/j.biochi.2011.05.009

[15] Tyler MJ, Stone DJM, Bowie JH (1992) A novel method for the release and collection of dermal, glandular secretions from the skin of frogs. J Pharmacol Toxicol Methods 28: 199–200. 1296824 10.1016/1056-8719(92)90004-k

[16] Almaaytah A, Zhou M, Wang L, Chen T, Walker B, et al (2012) Antimicrobial/cytolytic peptides from the venom of the North African scorpion, *Androctonus amoreuxi*: biochemical and functional characterization of natural peptides and a single site-substituted analog. Peptides 35: 291–299. 22484288 10.1016/j.peptides.2012.03.016

[17] Chen T, Orr DF, Bjourson AJ, McClean S, O'Rourke M, et al (2002) Novel bradykinins and their precursor cDNAs from European yellow-bellied toad (*Bombina variegata*) skin. Eur J Biochem 269: 4693–4700. 12230583 10.1046/j.1432-1033.2002.03174.x

[18] Rinaldi AC (2002) Antimicrobial peptides from amphibian skin: an expanding scenario. Curr Opin Chem Biol 6: 799–804. 12470734 10.1016/s1367-5931(02)00401-5

[19] Li R, Wang H, Jiang Y, Yu Y, Wang L, et al (2012) A novel Kazal-type trypsin inhibitor from the skin secretion of the Central American red-eyed leaf frog, *Agalychnis callidryas* . Biochimie 94: 1376–1381. 22464955 10.1016/j.biochi.2012.03.009

[20] Wang H, Wang L, Zhou M, Yang M, Ma C, et al (2012) Functional peptidomics of amphibian skin secretion: A novel Kunitz-type chymotrypsin inhibitor from the African hyperoliid frog, *Kassina senegalensis* . Biochimie 94: 891–899. 22197669 10.1016/j.biochi.2011.12.008

[21] Wang M, Wang L, Chen T, Walker B, Zhou M, et al (2012) Identification and molecular cloning of a novel amphibian Bowman Birk-type trypsin inhibitor from the skin of the Hejiang Odorous Frog; *Odorrana hejiangensis* . Peptides 33: 245–250. 22285790 10.1016/j.peptides.2012.01.003

[22] Liu L, Burcher E (2005) Tachykinin peptides and receptors: putting amphibians into perspective. Peptides 26: 1369–1382. 16042977 10.1016/j.peptides.2005.03.027

[23] Spindel ER (2006) Amphibian Bombesin-like peptides. Handbook of Biologically- Active Peptides, In: Kastin AJ, Editor. Academic Press. 277–289.

[24] Conlon JM (1999) Bradykinin and its receptors in non-mammalian vertebrates. Regul Pept 79: 71–81. 10100919 10.1016/s0167-0115(98)00160-8

[25] Galas L, Raoult E, Tonon MC, Okada R, Jenks BG, et al (2009) TRH acts as a multifunctional hypophysiotropic factor in vertebrates. Gen Comp Endocrinol 164: 40–50. 19435597 10.1016/j.ygcen.2009.05.003

[26] Woll PJ, Rozengurt E (1988) [D-Arg^1^, D-Phe^5^, D-Trp^7,9^, Leu^11^]-substance P, a potent bombesin antagonist in murine Swiss 3T3 cells, inhibits the growth of human small cell lung cancer cells in vitro. Proc Natl Acad Sci USA 85: 1859–1863. 2450349 10.1073/pnas.85.6.1859 PMC279880

[27] Palmer RM, Ferrige AG, Moncada S (1987) Nitric oxide release accounts for the biological activity of endothelium-derived relaxing factor. Nature 327: 524–526. 3495737 10.1038/327524a0

